# Computer-Aided Whole-Cell Design: Taking a Holistic Approach by Integrating Synthetic With Systems Biology

**DOI:** 10.3389/fbioe.2020.00942

**Published:** 2020-08-07

**Authors:** Lucia Marucci, Matteo Barberis, Jonathan Karr, Oliver Ray, Paul R. Race, Miguel de Souza Andrade, Claire Grierson, Stefan Andreas Hoffmann, Sophie Landon, Elibio Rech, Joshua Rees-Garbutt, Richard Seabrook, William Shaw, Christopher Woods

**Affiliations:** ^1^Department of Engineering Mathematics, University of Bristol, Bristol, United Kingdom; ^2^School of Cellular and Molecular Medicine, University of Bristol, Bristol, United Kingdom; ^3^Bristol Centre for Synthetic Biology (BrisSynBio), University of Bristol, Bristol, United Kingdom; ^4^Systems Biology, School of Biosciences and Medicine, Faculty of Health and Medical Sciences, University of Surrey, Guildford, United Kingdom; ^5^Centre for Mathematical and Computational Biology, CMCB, University of Surrey, Guildford, United Kingdom; ^6^Synthetic Systems Biology and Nuclear Organization, Swammerdam Institute for Life Sciences, University of Amsterdam, Amsterdam, Netherlands; ^7^Icahn Institute for Data Science and Genomic Technology, Department of Genetics and Genomic Sciences, Icahn School of Medicine at Mount Sinai, New York, NY, United States; ^8^Department of Computer Science, University of Bristol, Bristol, United Kingdom; ^9^School of Biochemistry, University of Bristol, Bristol, United Kingdom; ^10^Brazilian Agricultural Research Corporation/National Institute of Science and Technology – Synthetic Biology, Brasília, Brazil; ^11^Department of Cell Biology, Institute of Biological Sciences, University of Brasília, Brasília, Brazil; ^12^School of Biological Sciences, University of Bristol, Bristol, United Kingdom; ^13^Manchester Institute of Biotechnology, The University of Manchester, Manchester, United Kingdom; ^14^Elizabeth Blackwell Institute for Health Research (EBI), University of Bristol, Bristol, United Kingdom; ^15^Department of Bioengineering, Imperial College London, London, United Kingdom; ^16^School of Chemistry, University of Bristol, Bristol, United Kingdom

**Keywords:** whole-cell models, synthetic biology, systems biology, multiscale models, bioengineering, biodesign

## Abstract

Computer-aided design (CAD) for synthetic biology promises to accelerate the rational and robust engineering of biological systems. It requires both detailed and quantitative mathematical and experimental models of the processes to (re)design biology, and software and tools for genetic engineering and DNA assembly. Ultimately, the increased precision in the design phase will have a dramatic impact on the production of designer cells and organisms with bespoke functions and increased modularity. CAD strategies require quantitative models of cells that can capture multiscale processes and link genotypes to phenotypes. Here, we present a perspective on how whole-cell, multiscale models could transform design-build-test-learn cycles in synthetic biology. We show how these models could significantly aid in the design and learn phases while reducing experimental testing by presenting case studies spanning from genome minimization to cell-free systems. We also discuss several challenges for the realization of our vision. The possibility to describe and build whole-cells *in silico* offers an opportunity to develop increasingly automatized, precise and accessible CAD tools and strategies.

## Introduction

Whole-cell models (WCMs) are state-of-the-art Systems Biology formalisms: they aim at representing and integrating all cellular functions within a unique computational framework, ultimately enabling a holistic, and quantitative understanding of cell biology (Tomita, 2001; Karr et al., 2015a). Quantitative and high-throughput *in silico* experiments generated from WCMs promise to significantly shorten the distance between hypothesis/design formulation and testing (Carrera and Covert, 2015).

While simplified models for specific cellular functions were first developed over 30 years ago [e.g., gene expression regulation (McAdams and Arkin, 1997), signaling (Morton-Firth and Bray, 1998) and metabolic pathways (Cornish-Bowden and Hofmeyr, 1991), cell growth (Shu and Shuler, 1989) and the cell cycle (Goldbeter, 1991; Tyson, 1991; Novak and Tyson, 1993)], the first WCM, the E-Cell model, was only derived in the 1990s for *Mycoplasma genitalium*, which has the smallest genome among freely living organisms (Tomita et al., 1999). The so-called virtual self-surviving cell (SSC) model is partially stochastic; it includes only a subset of protein-coding genes and enables dynamic simulations which encompass various subcellular processes, including enzymatic reactions, complex formation and substance translocation. In parallel, the first genome-scale metabolic models (GSMMs) were developed by Palsson’s group (Varma and Palsson, 1994) using flux balance analysis (FBA) in the 1990s.

More recently, hundreds of GSMMs have been reconstructed for different organisms, with an increasing number of represented genes (McCloskey et al., 2013; Yilmaz and Walhout, 2017; Mendoza et al., 2019). GSMMs have been complemented with a mathematical description of other processes, such as transcription, translation, and signaling (Lee et al., 2008; Thiele et al., 2009). Less than a decade ago a more complete, hybrid WCM, representing all genes and molecular functions known for an organism, was reported by Covert’s group (Karr et al., 2012). In this pioneering work, Karr and colleagues integrated 28 sub-models to represent one cell cycle of *M. genitalium*; each sub-model is represented with a distinct formalism, including ordinary differential equations (ODEs), FBA, stochastic simulations and Boolean rules.

Substantial research and effort are still needed to improve WCMs’ descriptive power and to increase the complexity of organisms they can represent. Developing a WCM is a challenging task, which requires the collection of extensive experimental data, integration of sub-cellular models and *in silico*/*in vivo* model validation. A complete WCM should ideally integrate multiscale interactions at the cellular level (Karr et al., 2012; King et al., 2016) while accounting for the overall cellular structure (Betts and Russell, 2007), the dynamic structure of molecular interactions (Noske et al., 2008; McGuffee and Elcock, 2010; Yu et al., 2016), and the spatial compartment of the subcellular components (Ander et al., 2004; Takahashi et al., 2005; Thul et al., 2017). Ensuring an accurate representation of all of the cellular processes across organisms of increasing complexity is highly challenging (Bouhaddou et al., 2018; Singla et al., 2018; Szigeti et al., 2018). It is therefore not surprising that, to date, only the *M. genitalium* and, very recently, *E. Coli* (Macklin et al., 2020). WCMs have been released, although several other WCMs are currently under development^1^. We refer the reader to recent efforts which provide an overview of the state-of-the-art in the development of WCMs (Goldberg et al., 2018; Feig and Sugita, 2019).

**FIGURE 1:**
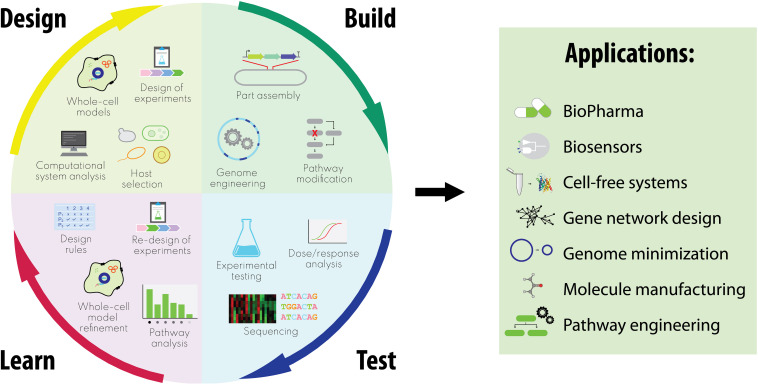
Integrated design-build-test-learn cycles in synthetic biology encompassing whole-cell model-guided approaches, and relative applications.

Here, we focus on the enormous potential we believe WCMs have for design-build-test cycles integrating synthetic with systems biology (Figure 1). While the applications are diverse, they share a high degree of complexity which would require extensive trial and error experimental cycles in the absence of robust computational design algorithms based on predictive models. We conclude by considering relevant challenges that must be addressed by interdisciplinary communities to fully realize our vision, discussing future directions for integrating WCMs through synthetic and systems biology.

## Whole-Cell Design Strategies in Synthetic Biology

### Model Granularity of Gene Network (re)Design

Mathematical models can be instrumental for the (re)design of network circuits that recapitulate definite biological functions. Knowledge of regulatory mechanisms in biological pathways has been gained by considering living systems as a composition of functional modules, which are investigated through minimal computer models. Examples include controllable oscillators (Marucci et al., 2009; Purcell et al., 2010, 2013; Tomazou et al., 2018), circadian clocks (Gerard et al., 2009; Ananthasubramaniam et al., 2020), signaling networks (Prescott and Abel, 2017), the metabolism (Castellanos et al., 2004; Pandit et al., 2017), and transcriptional regulation (Carrera et al., 2009). Existing minimal and detailed computer models span a broad range of granularity in their biochemical details. However, one may expect that, if the core design of a minimal and a detailed model is similar, their general properties will match.

The understanding of a living organism at a system’s level may be reached through decomposing it into functional modules or modular circuits (Hartwell et al., 1999; Kitano, 2002; Ravasz et al., 2002). The capability to sustain viability through autonomously generated offspring is essential. It is therefore a feature that WCMs shall account for through modeling cell division, which is intimately integrated with various layers of cellular regulation (metabolism, signaling, gene regulation, transcription, etc.). A number of minimal models have been developed for the eukaryotic cell cycle by Barberis’, Tyson’s and Novák’s groups (Battogtokh and Tyson, 2004; Barberis et al., 2012; Gerard et al., 2013, 2015; Linke et al., 2017; Mondeel et al., 2020).

Currently, the majority of multiscale models (not WCMs) lack components able to bridge cellular networks or function (cell cycle, metabolism, signaling, gene regulation, etc.). Identification of hubs, i.e., elements with high connectivity in the cellular environment that integrate cellular networks, is a critical feature of WCMs. Transcription factors have recently been identified as hubs that integrate multiscale networks, potentially connecting the cell cycle to metabolism (Mondeel et al., 2019), and can be among the parts of a system that influence its state as a whole. Multiscale frameworks coupling networks of differing granularity are being developed, by identifying the relevant regulations occurring among common network nodes and through the use of different mathematical formalisms (van der Zee and Barberis, 2019). These and other strategies are also being developed to integrate networks of cellular functional modules (Prescott et al., 2015). Together with the identification of networks underlying the cell’s autonomous oscillations, these strategies can rationalize the proper timing of offspring generation accounted by WCMs.

Designing synthetic gene networks by modeling and integrating them within WCM formalisms [as in Purcell et al. (2013)] could be critical to investigate how gene expression correlates with codon usage, explore possible cell burden effects (Borkowski et al., 2016), and predict modularity of synthetic gene networks and tools to modulate gene expression across different chassis (Way et al., 2014; Pedone et al., 2019; Gomide et al., 2020).

### Design and Engineering of Reduced Genomes

Minimal genomes can be defined as reduced genomes containing only the genetic material which is essential for a cell to reproduce (Glass et al., 2017). Studying and engineering minimal genomes can be instrumental both to understand the most essential tasks a cell must perform to sustain life, and to obtain optimal chassis for synthetic biology applications, with reduced cell burden and superior robustness (Moya et al., 2009; Hutchison et al., 2016; Ceroni and Ellis, 2018; Mol et al., 2018; Landon et al., 2019).

Exhaustive experimental characterization of a minimized genome is unfeasible: even for an organism as small as *M. genitalium* (0.58 mb and 525 genes), there are thousands of possible combinations of gene knockouts to be performed. Of note, this figure is most probably underestimated, accounting for the fact that the order in which gene deletions are performed can alter the resulting phenotypes (Gawand et al., 2015). Genome-scale computational models of cells could be instrumental to fully understand the dynamic and context-dependent nature of gene essentiality (Rancati et al., 2018), and to rationally design minimized genomes *in silico*. Computer-aided minimal genome engineering could significantly reduce the time and cost to reduce genomes compared to current approaches based on extensive experimental iterations (Posfai et al., 2006; Iwadate et al., 2011; Hirokawa et al., 2013; Hutchison et al., 2016; Zhou et al., 2016; Reuss et al., 2017; Breuer et al., 2019).

To the best of our knowledge, two top-down genome reduction approaches have been proposed so far based on genome-scale models. The MinGenome algorithm applies a mixed-integer linear programming (MILP) algorithm to a GSMM of *Escherichia coli*, using information pertaining to essential genes and synthetic lethal pairs within the optimization (Wang and Maranas, 2018). In contrast, Minesweeper and GAMA are top-down genome minimization algorithms based on the *M. genitalium* WCM. They exploit a divide-and-conquer approach and a biased genetic algorithm, respectively, to iteratively simulate reduced genomes (Rees-Garbutt et al., 2020); their *in silico* predictions have not been tested in the laboratory yet.

GSMM-based genome reduction algorithms such as MinGenome or analogous, adaptable metaheuristic techniques [e.g., (Burgard et al., 2003; Tang et al., 2015; Mutturi, 2017)] are currently more broadly applicable across organisms given the large availability of these formalisms. Still, as more WCMs become available, we expect WCM-based genome reduction algorithms to provide superior predictions of cellular processes and genetic interactions, thanks to their richness of multiscale cellular process representation.

### Design and Prototyping of Cell-Free Systems

Cell-free transcription/translation systems, based on crude cellular extracts, are a valuable platform to address fundamental biological questions in a controllable and reproducible way. In recent years, the decrease of costs associated with this technology and significant improvements in synthesis yield capabilities (Calhoun and Swartz, 2005) have made cell-free systems increasingly popular in synthetic biology for the prototyping and testing of engineered biological parts (McCloskey et al., 2013; Reuss et al., 2017; Yilmaz and Walhout, 2017; Mendoza et al., 2019) and networks (Noireaux et al., 2003; Siegal-Gaskins et al., 2014; Takahashi et al., 2015). As the possible applications of cell-free systems grow [see (Silverman et al., 2020) for a recent review], mathematical models are being developed to quantitatively formalize how biological processes perform within cell-free platforms (Koch et al., 2018).

So far, deterministic models (ODEs, or constraint-based) have been proposed to describe specific processes within cell-free platforms such as transcription and translation (Karzbrun et al., 2011; Stogbauer et al., 2012; Siegal-Gaskins et al., 2014), resource competition (Underwood et al., 2005; Borkowski et al., 2018; Matsuura et al., 2018; Moore et al., 2018), and metabolism (Vilkhovoy et al., 2018). The integration of mathematical formalisms across scales for cell-free platforms, building toward WCMs, could be highly beneficial to both facilitate *de novo* design of circuits, and to quantitatively compare *in vitro* cell-free products with their *in vivo* counterparts.

### Whole-Cell Biosensor Design and Testing

Biosensors are analytical devices which can convert a biochemical reaction into a measurable signal. The recognition unit in a biosensor can be composed of whole cells, nucleic acids, enzymes, proteins, antibodies or combinations thereof. Synthetic biology has significantly accelerated biosensor development; new generation whole-cell biosensors (i.e., sensors implemented throughout living cells) have been engineered, allowing, for example: arsenic detection (Diesel et al., 2009), detection of pollutants and antibiotics (van der Meer and Belkin, 2010), microbial detection in industrial settings (Lu et al., 2013) and *in vivo* diagnostic applications [e.g., detection of environmental signals in the gut (Kotula et al., 2014) and diagnosis of liver metastases (Danino et al., 2015); see (Slomovic et al., 2015) for an overview].

The application of WCMs to the design, prototyping and testing of whole-cell biosensors could suggest rational approaches to tune their sensitivity, stability, and dynamic range while facilitating the choice of the ideal chassis and, if needed, guide its re-engineering to optimize biosensor performance (Hicks et al., 2020). If WCMs become available for different chassis and entire organisms, they could also support the design of optimized targeted delivery of genetically encoded biosensors.

### Industrial Implications of Whole-Cell Models

Although the intellectual merit of pursuing a computer-aided whole-cell design approach is unquestioned, it is clear that the success of this endeavor will ultimately be judged by its impact on science, medicine, and industry. The increasing drive of computer-aided designs (CADs) toward “green” chemistry approaches, allied to increases in gene synthesis speed and capability and associated cost reductions, are making biosynthesis an increasingly appealing route for the manufacture of high-value chemicals (El Karoui et al., 2019). This includes a plethora of opportunities across the pharmaceutical, agrochemical, commodity chemical, and materials sectors, amongst others.

A major challenge, however, remains the development of robust, scalable microbial chassis, whose metabolic processes can be predictably tuned for a desired outcome (Xu et al., 2020). Currently, chassis choice is largely restricted to a subset of genetically tractable microorganisms, whose physiology and performance during fermentation are well understood, and for whom effective molecular genetic tools required for their manipulation exist. Chassis optimization to date has relied exclusively on incremental, stepwise improvements in desired host strain characteristics, including growth rate, feedstock utilization, and product yield (Calero and Nikel, 2019). For these reasons, the process of chassis optimization remains prohibitively slow and expensive, accounting in part for the paucity of high-value small molecules that are currently manufactured using synthetic biology processes. Targeted manipulations often lead to unanticipated off-target effects, linked to the co-dependency of metabolic processes, which generally function in concert within interdependent cellular networks (Woolston et al., 2013): perturbations may compromise rather than enhance desirable characteristics, leading to undesired outcomes. Clearly, robust, predictable WCMs represent an attractive solution to the problem of chassis optimization, affording a catch-all tool that can be used to unpick dependencies and ensure that performance criteria can be met.

Additionally, the complexities associated with population heterogeneity during chassis fermentation must be resolved (Danchin, 2012). For fermentation-based industrial processes to be tractable, product yields must be sufficiently high to make biosynthesis financially viable. The emergence of “cheaters” or slow-growers within microbial populations should be tackled with tunable regulatory processes that operate throughout populations. The introduction of such characteristics is a major challenge to conventional chassis design approaches. WCM-driven approaches could more easily implement and test these processes.

Critical to the success of a computer-aided whole-cell design approach is the quality of the employed model (Fernandez-Castane et al., 2014). Microbial systems with small genomes represent a compelling entry point for study, with model development possibly being facilitated by ongoing studies focused on establishing the core constituents of a functional genome. These studies are in part driven by genome minimization experiments, which in turn can be used to further refine model performance. Importantly, fundamental gaps remain in our understanding of microbial metabolic processes, and this will unquestionably hinder progress (Price et al., 2018). However, the capacity of WCMs to predict previously unidentified metabolic dependencies should be viewed as an acid test of model validity. Indeed, GSMMs often fail due to their inability to account for metabolic dependencies, a feature which has led to skepticism within industrial circles, questioning the value of such models. Whole-cell approaches offer a mechanism to circumvent this issue. This is of particular significance when developing chassis for “non-natural” products whose chemistries sit outside those of metabolites found in nature (Calero and Nikel, 2019). Expanding the metabolic capacity of chassis organisms to deliver such novel products risks introducing additional complexities, including excessive depletion of core metabolite pools or the generation of toxic products or intermediates. Design approaches driven by WCMs are uniquely placed to identify such issues and provide a route to their circumvention.

The capacity to design-in explicit control over cellular behavior is also critical for industrial adoption of model-derived chassis. It can be argued that the ability to regulate cellular processes is as important as defining the processes themselves. Tunable regulatory systems must afford a degree of both intrinsic and extrinsic control. Synthetic biology-based approaches for constructing genetic circuitry are now placing us on a path to broad-reaching cellular regulation, though issues still exist. These systems are often insufficiently orthogonal, with bespoke designs required for different chassis due to variations in core metabolic process (Pandit et al., 2017). Again, whole-cell design approaches offer a solution to this issue, as such systems can be predefined and tested for functionality *in silico* prior to undertaking costly lab experimentation.

## What’s Next? Going Beyond the Prototype

In recent years, advances in genomic measurement technologies for data generation, the establishment of data repositories, and the development of WCM simulation platforms have significantly facilitated the derivation of WCMs [see (Goldberg et al., 2018) for a review]. Nevertheless, the implementation of WCM-based design-build-test cycles for genome-scale engineering requires further challenges to be addressed (Bartley et al., 2020).

If a model has to be used for the design and prototyping of an engineered living system, the model needs to be reliable. Even for a simple organism, the number of kinetic parameters raises as the complexity and the level of detail of a mathematical model increase; constraining parameters thus becomes harder and requires extensive experimental data. Mathematical models can be used to produce predictions of missing data, however, they often abstract physical processes using simplifying assumptions which might hold in specific conditions (Babtie and Stumpf, 2017). To set the 1,462 quantitative parameters of the *M. genitalium* WCM, values from related organisms were incorporated due to a lack of organism-specific data (Macklin et al., 2014); a combination of parameter values reported from previous experiments and numerical optimization on a reduced model was performed. While, ideally, we would like to measure all kinetic parameters directly from experiments, we still lack the ability to measure each state in individual cells over time, and across all possible environmental conditions. A combination of direct experimental estimation and parameter inference will likely be needed for genome-scale formalisms and WCMs.

Sensitivity analysis, usually performed by perturbing parameters to understand how uncertainties affect the model outputs (Erguler and Stumpf, 2011), can become extremely computationally expensive when applied to genome-scale models. Alternatively, statistical approaches such as those based on Bayesian methods (Vernon et al., 2018) or the Fisher information matrix (Rand, 2008) could be carefully carried out at least at the sub-model level, and possibly scaled up to WCMs. The Reverse Engineering Assessments and Methods (DREAM8) parameter estimation challenge (Karr et al., 2015b) was organized to develop new parameter estimation techniques specific for WCMs. It suggested possible interesting avenues for WCM parameterization (i.e., model reduction and a combination of differential evolution and random forests), and highlighted that the availability of comprehensive data is critical to ensure the model is practically identifiable (Ashyraliyev et al., 2009), and to calibrate WCMs.

Researchers have started to collect data needed for WCM development into public repositories [e.g., (Wittig et al., 2012; Kolesnikov et al., 2015; Sajed et al., 2016; UniProt Consortium, 2018; Caspi et al., 2020)]; still, the data needed to derive and fit WCMs are dispersed across many repositories and publications and often not annotated or normalized, ultimately requiring a massive manual effort. Federated archives of repositories, such as the PDB-Dev system to deposit Integrative/Hybrid models and corresponding data (Burley et al., 2017), also exist and might be well placed to archive and disseminate both data and models, while enabling different researchers to attempt alternative modeling/parameterization approaches. Covert’s group developed the WholeCellKB database (Karr et al., 2013) to organize the quantitative measurements (over 1,400) from which the *M. genitalium* WCM was derived; it would be ideal to enable automatic access and querying in such databases.

To enhance WCM reproducibility and collaboration, new standards and simulations software are also needed (Medley et al., 2016). Researchers should invest efforts to use and expand the capabilities of standard formats such as the Systems Biology Markup Language (SBML) (Hucka et al., 2003) and the Systems Biology Graphical Notation (SBGN) (Le Novere et al., 2009) to be suitable for WCMs. For example, several aspects of the *M. genitalium* WCM cannot be represented by SBML, such as the multi-algorithmic nature of the model (Waltemath et al., 2016). Further development of standard modeling formats is needed to enable reproducible WCM simulations, e.g., by including in the SMBL Hierarchical Model Composition package ontologies which could represent the algorithm needed for specific sub-models (Courtot et al., 2011). In the context of synthetic biology applications, we believe it would be appropriate and beneficial to report and deposit data related to various iterations of WCM-generated *in silico* predictions, *in vivo* testing and possible model/design refinement; this would establish the predictive power of WCMs and illuminate steps to make design-build-test-learn cycles more effective.

It is also important to consider the structural uncertainties in the model, which depend on model assumptions. While, for certain sets of models (e.g., small ODE systems for signaling pathways), likelihood- and Bayesian-based approaches have been proposed for model selection (Wilkinson, 2007; Kirk et al., 2013) and semidefinite programming for model invalidation (Anderson and Papachristodoulou, 2009), no suitable techniques for WCMs have been proposed to date.

We foresee that automation will play a fundamental role in the derivation of WCMs for eukaryotic organisms and in their application to design complex processes. Ideally, we would like to introduce automation at different stages, such as data extraction from the literature, model derivation, and model/data integration both within the model fitting and validation steps, and when comparing *in silico* design prediction with *in vivo* tests (Bartley et al., 2020). This, in turn, will require the adoption of standards for both data and model repositories. Also, laboratory automation, coupled to WCM-based CAD, is expected to transform design-build-test cycles. As the use of robotics becomes increasingly common in both academia and industry, the throughput and reproducibility of experiments needed for both WCM derivation and validation can be significantly increased, and protocol sharing across research communities facilitated (Jessop-Fabre and Sonnenschein, 2019).

To assist the adoption of WCMs for synthetic biology applications, high-performance parallelized computer clusters are required to run the models with lengthy runtimes, coordinate the corresponding databases, parameterize and validate the models, and then to integrate WCMs in design cycles in combination with optimization algorithms (Macklin et al., 2014; Chalkley et al., 2019).

The implementation of standardized tools to share data and simulate WCMs would, in turn, facilitate model validation. This should involve the definition of proper metrics and formal model verification techniques such as those developed for SBML-encoded models (Kwiatkowska et al., 2011).

## (re)Thinking System Approaches: A Collaborative Effort

In addressing the aforementioned challenges, we believe there is a tremendous opportunity to rethink approaches used so far to generate genome-scale models, including WCMs, and to integrate with broader communities including software engineers, computer scientists, structural biologists, bioinformaticians, and systems and synthetic biologists.

We do anticipate that, as diverse communities synergize on WCM-related research, different kinds of formalisms might be integrated within genome-scale models. Symbolic reasoning provides a range of expressive and intuitive logical frameworks that could potentially complement and help glue together sub-models at different scales. Such methods are routinely applied to complex systems in the electronics and software industries, and have been applied to biological systems for nearly a decade (Iyengar, 2011). Recent work showed the feasibility of applying logic programming methods to signaling pathways (Ray et al., 2011), metabolic networks (Bragagli and Ray, 2015) and automating a mechanistic philosophy of scientific discovery in simulated organisms (Rozanski et al., 2015); it should be feasible to integrate such sub-models within a WCM framework.

We believe there is scope to further increase the descriptive and predictive ability of WCMs across spatial and temporal scales by integrating the structural biology and the molecular modeling communities to carefully consider not only the biochemical, but also the physical, molecular and structural components of cells. The development of the so-called “physical” WCMs [see (Feig and Sugita, 2019) and (Feig and Sugita, 2013) for comprehensive reviews] is an emerging field, with the first models describing minimal cellular environments in full atomistic detail (Feig et al., 2015; Yu et al., 2016). With the final aim to integrate biochemical and physical WCMs within a multiscale framework (Sali et al., 2015), we need approaches which can cope with the limitations of atomistic models of biomolecules (mainly in terms of computational resources), possibly exploiting coarse-grained (Ando and Skolnick, 2010; Hyeon and Thirumalai, 2011) or continuum (Solernou et al., 2018) approaches.

By collaborating with software engineers, we need to develop tools which can enable, and possibly automate, the integration of different data types across scales, model derivation, fitting and validation, and visualization and interpretation of results (Szigeti et al., 2018).

Moreover, rule-based models might become the new standard to represent each molecular species with the required level of granularity and multi-algorithmic sub-models (e.g., FBA and stochastic dynamical models). Frameworks where intuitive logic is coupled to rule-based models have started to be developed recently (van der Zee and Barberis, 2019).

As we produce ever-increasing amounts of experimental data and increasingly sophisticated computational tools to realize detailed and complex representations of actual cells, approaches instead focusing on deliberately abstract and parsimonious simulations of artificial cellular systems provide a valuable change of perspective. Such “toy models” might be a valuable tool to test different algorithms for model derivation and fitting, while offering an opportunity to engage with broader research communities and with the public (Castiglione et al., 2014).

Finally, we believe there is tremendous potential for applying machine learning techniques to both WCM derivation and their applications in synthetic biology. Two recent works (Lin et al., 2017; Ma et al., 2018) showed that deep neural networks are well placed to reconstruct the architecture of living systems [namely, the hierarchical organization of nuclear transcriptional factors in the nucleus (Lin et al., 2017) and of a basic eukaryotic cell (Ma et al., 2018)] and predict cell states and phenotypes. In both cases, the configuration of network layers and thus the biological structure were formulated using extensive prior knowledge, ultimately enabling fully “visible” systems, where all the internal biological states can be interrogated mechanistically (Yu et al., 2018). Machine learning could be beneficial to systematically process large *in vivo* and *in silico* whole-cell data-sets, for example by applying Bayesian inference, to integrate data from diverse sources and supplement sparse data (Perdikaris and Karniadakis, 2016), and to help to automatically classify WCM simulations and link phenotypes to genotypes (Alber et al., 2019). Ensemble methods, which combine multiple independent models into a single predictive model for increasing the overall robustness of predictions, might also be adopted to develop subcellular formalisms and support their integration across chassis (Camacho et al., 2018). Additionally, machine learning might assist in WCM parameter identification, for example applying Bayesian parameter estimation (Vyshemirsky and Girolami, 2008), regression models and reinforcement learning techniques (Alber et al., 2019). Optimal experimental design techniques might also offer a valuable methodology to select the best experimental datasets for both model identification and validation (Smucker et al., 2018).

## Discussion

We have shown that WCMs are likely to be instrumental to inform design-build-test cycles across synthetic biology applications. WCMs can accelerate the realization of “designer” cells and organisms tailored to specific functions, reducing experimental iterations and increasing the predictive power of computational formalisms used so far.

In the (re)design of cellular network functionalities, it is therefore important to quantitatively analyze and predict, through dedicated modeling strategies, the dynamics of interactions between various layers of cellular regulation. Thus, WCMs should take into account how different cellular layers are integrated, and how regulatory feedback among these layers occurs in time. These challenges must be tackled through integrative computational and experimental collaborative efforts aimed, respectively, toward: (i) engineering *in vivo* network designs which, through predictive systems biology, may be able to autonomously oscillate, sustaining generation of offspring, and (ii) extraction, visualization and functional exploration of regulatory interactions among cellular layers through novel multiscale modeling frameworks.

As synthetic biology moves toward the (re)engineering of entire genomes and multicellular systems, interdisciplinary communities need to collaborate for the development of tools that are required to improve the predictive power of WCMs. Although challenges remain, it is clear that the adoption of model-based methods has the potential to transform both basic research and the current bioproduction development process, leading to marked improvements in host performance and product yield on an industrial scale.

Ultimately, as the development of human genome-scale kinetic models becomes more feasible (Bordbar et al., 2015; Szigeti et al., 2018), we anticipate that whole-cell formalisms will become an indispensable tool to study human variation, and design treatments and synthetic cellular screening systems.

## Author Contributions

LM, MB, JK, OR, and PR wrote the manuscript. MS prepared the figure. All other authors participated to discussion within the workshop, helped with editing, and/or provided feedback.

## Conflict of Interest

The authors declare that the research was conducted in the absence of any commercial or financial relationships that could be construed as a potential conflict of interest.

## Footnotes

1 https://www.wholecell.org/
